# Evaluation of acute/late toxicity and local recurrence in T1–T2 glottic carcinoma treated with accelerated hypofractionated 3D-conformal external beam radiotherapy (3D-CRT)

**DOI:** 10.2478/raon-2013-0020

**Published:** 2013-05-21

**Authors:** Vassilis E. Kouloulias, Anna Zygogianni, Eftychia Mosa, Kalliopi Platoni, John Georgakopoulos, Christos Antypas, Ivelina Beli, Maria Tolia, Paulos Maragoudakis, Ioannis Giotakis, Zisis Papas, Amanda Psyrri, Nikolaos Kelekis, John Kouvaris

**Affiliations:** 1 2^nd^ Department of Radiology, Radiotherapy Unit, Attikon University Hospital, Medical School, Athens, Greece; 2 1^st^ Department of Radiology, Radiotherapy Unit, Aretaieion Univeristy Hospital, Medical School, Athens, Greece; 3 2^nd^ Otorhinolaryngology Clinic, Attikon University Hospital, Medical School, Athens, Greece; 4 Otorhinolaryngology Clinic, Thriasion Hospital, Athens, Greece; 5 Medical Oncology Unit, Attikon University Hospital, Athens, Greece

**Keywords:** early glottic cancer, accelerated hypofractionated radiotherapy, toxicity, local recurrence

## Abstract

**Background:**

The aim of the study was to evaluate the efficacy, as well as the acute and late toxicity of an accelerated hypofractionated 3DCRT schedule as radical treatment in patients with organ confined glottic cancer cT1-2N0.

**Patients and methods.:**

Between June of 2004 and September 2010, 47 retrospectively selected patients (29 males, 18 females) diagnosed with organ confined T1 or T2 glottic cancer, were treated with external 3DCRT in an accelerated hypofractionation schedule. The median age was 70 years. A dose of 64.4 Gy in 28 daily fractions was prescribed. The primary study endpoints were to assess the acute and late effects of radiation toxicity, according to the EORTC/ RTOG scale, as well as the therapeutic impact of this schedule in terms of local recurrence.

**Results:**

The median follow up was 36 months. At the end of radiotherapy, grade I, II and III acute toxicity was observed in 34, 9 and4 patients, respectively. Late grade I and II toxicity was observed in 25 and in 8 patients respectively. Only two local recurrences were observed, 15 and 24 months post 3DCRT respectively.

**Conclusions:**

Our radiotherapy schedule achieves a high locoregional control rate with the advantage of voice preservation. The proposed hypofractionated schedule can be recommended as a standard radiotherapy treatment, since these results are comparable with those of conventional fractionation schedules.

## Introduction

Cancer of the larynx is the most common cancer of the head and neck region. Risk factors include tobacco, alcohol, betel and areca nuts and deficiencies of iron, vitamin B12 and vitamin C.^1^

Early stage (T1 or T2) glottic cancer can be successfully treated with either radical radiotherapy or laser surgery.^2^ Possible treatment options in T1N0M0 disease include external beam radical radiotherapy, cordectomy, endoscopic resection (with or without laser) and partial laryngectomy.^3^ In T2N0M0 glottic cancer, definitive radiotherapy remains a valid treatment option with the benefit of voice preservation.^4^ It is usually preferred to other treatment modalities because of the high cure rates and the associated less impairment of vocal function.^5^ Surgery is usually employed after radiation as the salvage treatment in the event of locoregional relapse. Taking into account also, that a shorter hypofractionated schedule for the treatment of localized glottic cancer, will allow some sparing in radiotherapy resources in general, accelerated hypofractionated schemes are a good alternative to conventional radiotherapy fractionation.^6^

In our University we have established the 2.3 Gy per fraction as a standard treatment from 1997 when we also initially reported on the efficacy of accelerated hypofractionated radiotherapy for glottic carcinoma.^7^ With this current study we are reporting our experience in terms of the treatment outcome (response and toxicity).

## Patients and methods

### Patients’ characteristics

Between June of 2004 and September 2010, forty-seven patients with early stage larynx cancer (T1N0 = 38; T2N0 = 9) were retrospectively entered to this study. Twenty nine patients were males and eighteen were females. The median age at the time of the diagnosis was 70 years (range: 57–81). All patients had a good performance status according to Eastern Cooperative Oncology Group performance score of 0–1. The patients’ characteristics are summarized in Table 1.

The pretreatment evaluation at presentation included pathology review, laryngoscopy, biopsy of the tumour, laboratory studies with complete blood count, chemistries and radiological imaging of CT and/ or MRI of the head and neck to confirm the disease stage. Eligible patients had histologically confirmed localized glottic cancer with clinical stage (cT1-2 N0) (according to American Joint Committee on Cancer staging manual, 7^th^ edition, 2010) and the histologic type of the malignancy was that of an invasive squamous cell carcinoma. Patients were excluded if they had a history of previous radiotherapy in head and neck region, if they were suffering from a concurrent secondary carcinoma and if they were treated for both cancers simultaneously.

The patients were referred either to ATTIKO University Hospital or to Aretaieion University Hospital of Athens in order to be treated with radical radiotherapy as the initial treatment. None of the patients received chemotherapy. All candidates had to sign an informed consent form, concerning the side effects of the hypofractionated irradiation schedule. The primary study endpoints were to assess the acute and late effects of radiation toxicity, according to the EORTC/RTOG scale, as well as the therapeutic impact of this schedule in terms of local recurrence.

### Radiotherapy treatment and radiobiological assessments

CT-scan images (3mm slice thickness) were acquired and transferred to the treatment planning system. Patients were treated in the supine position with neck extended while immobilized with a thermoplastic head mask. Patients were instructed not to move or swallow during CT scan and simulation, and during the whole course of treatment. The CT datasets were transferred either to the Prosoma® Virtual simulation or to Plato® contouring system, through a DICOM III network. All contouring of clinical target volume (CTV), planning target volume (PTV) and normal structures (organs at risk-OARs) was performed according to the International Commission on Radiation Units and Measurements (ICRU) criteria.

Dose calculations were performed using either the treatment planning system Eclipse (Varian Associates, Palo Alto, CA) or the PLATO (Nucletron, The Netherlands), to deliver the prescribed dose to the International Commission on Radiation Units and Measurements (ICRU) reference point.

We kept the dose range between 95% and 107% of prescribed dose. Wedge compensation was used to ensure a uniform dose distribution throughout the target volume. To evaluate the dose constraints for normal tissues we used the QUANTEC trial corrected for hypofractionation.^8^

We used linear-quadratic (LQ) modeling in order to equate the hypofractionation schedules to the Normalized Total Dose (NTD) if delivered in 2 Gy-fractions, while we also included the impact of time:^9^–^11^

$$BED=nd\left(1+\frac{d}{\alpha /\beta }\right)-0.693\frac{T-Tk}{\alpha Tpot}$$

where n = number of fractions; d = dose per fractions; T = total irradiation time in days; Tk = time when repopulation starts; Tpot = potential doubling time. Specific values for head and neck tumours: α/β = 10; the Tk = 21days; α = 0.35 Gy^−1^ and the Tpot = 5.

The equivalent dose by means of NTD to conventional schedule (35 x 2 Gy) is:

$$NTD_{tumor}=\frac{Dnew\left(\frac{\alpha }{\beta }+dnew\right)+\frac{\alpha }{\beta }0.396\left(Teq-21\right)-\frac{\alpha }{\beta }0.396\left(Tnew-21\right)}{\frac{\alpha }{\beta }+2}$$

where Dnew = 64.4 Gy; α/β = 10; dnew = 2.3 Gy; Teq = 46 days (conventional scheme) and Tnew = 38 days (accelerated scheme).

Thus, NTD represents the dose given in 2 Gy fractions that would give the equivalent biologic effect to the new hypofractionated dose, with a value of NTD_tumour_ = 68.65 Gy.

In case of calculations for late effect on normal tissues (OAR):

$$NTD_{OAR}=D_{new}^{OAR}\frac{d_{new}^{OAR}+\alpha /\beta }{2+\alpha /\beta }$$

where $D_{new}^{OAR}$ and $d_{new}^{OAR}$ are the total dose and dose per fraction, respectively, for the suggested hypofractionation scheme related to OARs, while the α/β for late effects regarding OARS was set to α/β = 3.^10^,^11^ Under the above conditions, NTD_OAR_ = 68.26 Gy.

The patients were treated with lateral opposed fields. The margins of these fields were set according to CTV and PTV delineation, usually with the superior border at the level of hyoid bone and the inferior border set at the inferior margin of the cricoid cartilage.

Weighted beams and wedges were used as necessary, to improve dose homogeneity. In general, the fields were placed isocentrically. Radiotherapy was delivered once daily 2.3 Gy per fraction, five times a week for a period of 28 days. For the treatment technique, histograms were generated and a number of parameters, including mean, median and maximum dose, were evaluated. Patient setup was monitored weekly using portal films.

Patients were treated with megavoltage equipment, either on a VARIAN CLINAC 600C Linac with 6 MV photons, or ELECTA 6MV Linac. Portal films with amorphous siliceous electronic image device were obtained in the treatment position with therapeutic beam to confirm adequate coverage.^12^

### Follow up

The patients were examined weekly during the treatment by indirect laryngoscopy and reviewed every month later on, after the radiotherapy, in order to assess acute/ late toxicities.

Symptoms occurring in the intervals between the start of radiotherapy and 90 days after this time point are classified as “acute”. “Late” radiation complications were defined as those appearing 3 months from the end of treatment. The evaluation of acute and late radiation induced toxicity was done with the EORTC/RTOG toxicity criteria. Median follow-up duration was 36 months (range: 15–96 months).

### Statistical analysis

The overall survival rate (OS) and local recurrence free survival (RFS) rates were calculated from the onset of the radiotherapy, using Kaplan-Meier method. The analysis was performed with the SPSS ver 10 software (IL, USA).

Event for OS was death related to the disease. Failure after radiotherapy was considered an event when calculating the RFS. The surgical control after radiotherapy was not considered in this study.

## Results

Nearly all patients completed the planned 3D-CRT. Forty five patients completed the irradiation schedule with 64.4 Gy in 28 daily fractions, while two patients failed to receive the whole treatment course with 28 fractions. These two patients received 26 fractions with 2.3 Gy per fraction, while they didn’t complete the scheme due to moderate acute effects (severe edema in the larynx).

### Assessment of tolerability and acute treatment-related toxicity

Forty three patients developed mucosal reactions during the treatment (mainly arytenoid oedema) that necessitated the treatment with non-steroidal drugs and corticosteroids. At the end of radiotherapy, grade I, II and III acute skin toxicity was observed in 34, in 9 and in 4 patients, respectively. There were no patients with severe (grade 4) reported toxicities. Late toxicity as grade I, II was observed in 25 and in 8 patients respectively. No patient experienced a severe late radiation reaction (grade III or more), like laryngeal edema that required tracheotomy after the completion of radiotherapy. In details, the acute and late toxicity score is shown in Table 2.

**FIGURE 1:**
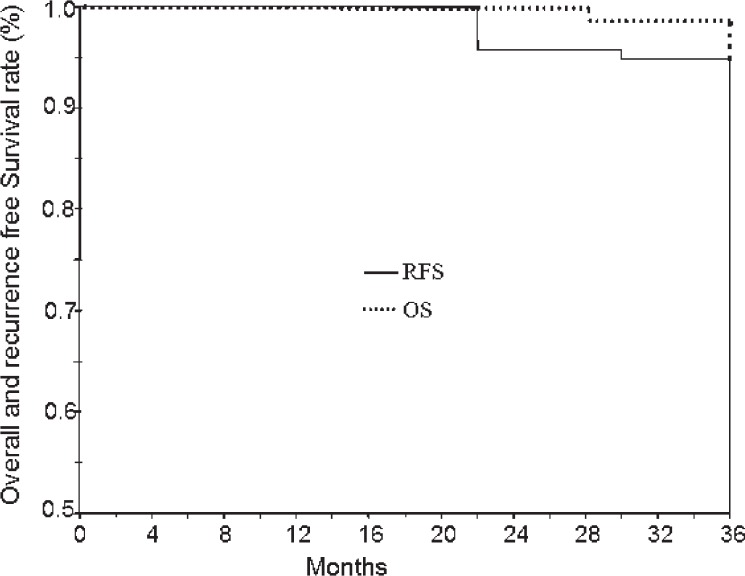
Recurrence free survival (RFS) and overall survival (OS) in patients with T1–2 glottic carcinoma.

The OS was 97.8% at 3 years. Figure 1 shows the Kaplan-Meier curve of OS and RFS, during follow-up. Only two local recurrences were observed 15 and 24 months post 3DCRT, respectively. Of the two patients that failed to complete the treatment, the first experienced relapse 15 months after radiotherapy and underwent surgical excision. He experienced a second relapse with cervical nodal metastatic disease, so he received radiotherapy, *via* intensity modulated radiotherapy (IMRT), but, unfortunately, he succumbed to his disease six months after re-irradiation.^13^ The second patient had a recurrence 24 months after radiotherapy and recently he completed the re-irradiation schedule, via IMRT. The RFS in general was 95.7% at 3 years.

## Discussion

Laryngeal cancer is the most common cancer of the head and neck region. Radiotherapy is an effective treatment for early laryngeal cancer with the advantage of larynx preservation. Reported 5-year control rates are 85–95% for T1N0 disease and 75–85% for T2N0 disease.^14^ Our study showed that RFS was 95.7% at 3 years and the overall survival was 97.8% at 3 years.

The acute toxicity reported was Grade I–II and in terms of mild hoarseness, erythema of the mucosa and less sore throat and cough requiring antitussive medication. The incidence of moderate and severe toxicity increased during the treatment, with a peak at the 6th week or irradiation, and then progressively decreased up to 3 months after the end of radiotherapy. The late toxicity observed was mainly hoarseness and arytenoids edema, but none of our patients experienced grade III–IV toxicity.

A number of prognostic factors like the T stage and impaired cord mobility have been well documented, while according to a number of recent studies, larger fraction sizes and shorter overall treatment times have led to improved outcomes. More specifically, the fraction size has proved to be the only significant predictor for the laryngectomy free survival (LFS).^15^,^16^

Some investigators have reported that a long overall treatment time in cases of T1–T2 glottic cancers, has a negative impact on the treatment outcome. Skladowski *et al*. reported that a 10-day prolongation of overall treatment time, from 45 days to 55 days, decreased the tumour control probability by 13%.^17^,^18^ Onimaru *et al*.^19^ reported that overall treatment time plays an important role in the clinical outcome for the glottic cancer treatment. The decrease of the treatment time proved to be useful for overcoming tumour repopulation and achieving a good local control for tumours that have a high a/β ratio and short potential doubling time. A large dose per fraction appeared to be beneficial because of the short overall treatment time, not because of any direct effects exerted by the large dose per fraction. The results of our study are in accordance with the relative results demonstrated from the study of Onimaru *et al*. More specifically, our report demonstrated that large doses per fraction and shorter overall treatment time succeeded a 97.8% survival rate and showed that RFS was 95.7% after a three years’ follow-up.

According to Short *et al*.^15^, actuarial local control, overall survival and LFS were assessed using Kaplan-Meier method and were measured from the date of starting radiotherapy to the date of the last follow-up or death. Potential predictors of outcome of death, disease relapse and laryngectomy were assessed by univariate and multivariate analyses. These predictors included sex, anterior commissure involvement, dose per fraction (≤ 2.25 *vs*. > 2.25 Gy), field size (< 25 *vs.* ≥ 25 cm^2^), overall treatment time (≤ 30 *vs*. >30 days) and tumour differentiation (well *vs*. poorly differentiated). The most significant prognostic factors for local control resulted to be the extent of the disease, the fraction size and the overall treatment time.

Apart from that, Duncan *et al*.^20^ reported a review, assessing the importance of treatment gaps on the outcome of radiotherapy for laryngeal cancer. A significance increase in local relapse rates was noted if treatment time exceeded 31 days and an increase in laryngeal cancer deaths if treatment time exceeded 30 days.

Additionally, Le *et al*.^21^ carried out a retrospective analysis of over 400 patients with T1 and T2 glottic cancer, assessing 15 potential prognostic factors influencing local control. On multivariate analysis, fraction size had a significant effect on local control in T2 group along with total dose, overall treatment time, treatment era, cord mobility and subglottic extension. Fraction size and overall treatment time were significant in T1 disease on the univariate analysis only.

Yu *et al*.^22^ assessed the effect of fraction size in a large retrospective review of patients with T1 glottic cancer treated with differing dose per fraction over a 10-year period, 1978–1988. Patients treated with >2 Gy per fraction, had an improvement in local control at 84%, compared with 65.6% for 2 Gy per fraction at 84-month follow-up. Fraction size was the only significant predictor of the outcome on univariate and multivariate analyses.

Gowda *et al*.^23^ reviewed 200 patients with T1 glottic cancer from the Christie and Royal Marsden Hospitals treated with a 3-week regimen delivering 50–52.5 Gy in 16 fractions. Their report mentioned an excellent 5-year local control rate (93%) and cause–specific survival (97%) with minimal toxicity. Furthermore, the study reported that overall treatment time and large fraction size may be advantageous in this group of patients with laryngeal cancer.

Hypofractionated radiotherapy in general has had great appeal as a strategy for improving the therapeutic gain of patients treated with radiotherapy and especially for patients with head and neck cancer. In line with these studies, our report also demonstrated the efficacy and safety of hypofractionated radiotherapy in early glottic cancer. At the same time, we showed that a 3DCRT hypofractionated schedule up to 64.4 Gy, with a 2.3 Gy daily fraction, not only managed to succeed high proportion rates of overall survival (97.8%), RFS (95.7%) and preservation of the voice, but also gave the opportunity of a treatment to patients that are unable to visit the hospital for a long period of time. Additionally, hypofractionated radical radiotherapy of early glottic cancer may be beneficial for the logistics of a hospital, succeeding a shorter radical treatment schedule and consequently a shorter patients’ waiting list.

Agarwal *et al*.^24^ reported that hypofractionation did not show any negative impact and that there is a considerable improvement of voice quality following radiotherapy. According to Di Nicola *et al*.^25^ in their institution study, 40 patients with T1 squamous cell carcinoma of the true vocal cord were irradiated with curative intent. The results of the report showed that although all patients before radiotherapy had an overall voice quality deteriorating, the vocal performance was strongly improved at 36 months after irradiation. Thus, there was a significant difference between pre- and post-radiotherapy for both groups.

The studies of Cheah *et al*. and Kazi *et al*., enhance the result that hypofractionation for T1N0M0 larynx carcinoma offers high locoregional control rates with voice preservation.^6^,^26^

## Conclusions

External radiotherapy is an effective treatment for early glottic carcinomas. Our retrospective study demonstrated that 3D-CRT hypofractionation up to 64.4 Gy in 28 daily fractions, is a feasible and safe modality, achieving improved local control rates, overall survival, laryngectomy free survival, decreased levels of toxicity, while at the same time preserves and improves the quality of voice. At last but not least, it seems that the accelerated schedule of 3DCRT with 2.3 Gy per fraction is a good alternative for radiotherapy departments with high workload. However, a randomized study is needed to confirm our results: due to the retrospective nature of our study, the small number of evaluated patients, and selection of patients included should be considered as preliminary.

## Figures and Tables

**Table 1:** Patients Characteristics (n=47)

**Parameter**	**Patients**
**Age**	
Median	70
Range	57–81
**Sex**	
Male / female	29/18
**Tumor**	
T1	38
T2	9

**TABLE 2:** Acute and late radiation induced skin toxicity according to EORTC/RTOG criteria

		**Patients**	**Percentage**
**Acute toxicity**			
Grade 0	None	0	
Grade 1	Mild or intermittent hoarseness	34/47	72.3%
	Cough not requiring antitussive		
	Erythema of mucosa		
Grade 2	Persistent hoarseness but able to vocalize	9/47	19.1%
	Referred ear pain, sore throat, patchy fibrinous exudate or mild arytenoid edema not requiring narcotic		
	Cough requiring antitussive		
Grade 3	Whispered speech, throat pain or referred ear pain requiring narcotic	4/47	8.5%
	Confluent fibrinous exudate, marked arytenoid edema		
Grade 4	Marked dyspnea, stridor or hemoptysis with tracheostomy or intubation necessary	0	
**Late Toxicity**			

Grade 0	None	14/47	29.8%
Grade 1	Hoarseness	25/47	53.2%
	Slight arytenoid oedema		
Grade 2	Moderate arytenoids oedema	8/47	17%
	Chondritis		
Grade 3	Severe oedema	0	0
	Severe chondritis		
Grade 4	Necrosis	0	0
